# Relationship between *Toxoplasma gondii* infection and psychiatric disorders in Iran: A systematic review with meta-analysis

**DOI:** 10.1371/journal.pone.0284954

**Published:** 2023-08-08

**Authors:** Mahbobeh Montazeri, Elahe Moradi, Mahmood Moosazadeh, Seyed Hamzeh Hosseini, Mahdi Fakhar

**Affiliations:** 1 Toxoplasmosis Research Center, Communicable Diseases Institute, Mazandaran University of Medical Sciences, Sari, Iran; 2 Student Research Committee, Mazandaran University of Medical Sciences, Sari, Iran; 3 Gastrointestinal Cancer Research Center, Non-communicable Diseases Institute, Mazandaran University of Medical Sciences, Sari, Iran; 4 Psychiatry and Behavioral Sciences Research Center, Mazandaran University of Medical Sciences, Sari, Iran; 5 Iranian National Registry Center for Toxoplasmosis (INRCT), Imam Khomeini Hospital, Mazandaran University of Medical Sciences, Sari, Iran; State University of New York Upstate Medical University, UNITED STATES

## Abstract

**Background:**

*Toxoplasma gondii*, a ubiquitous parasitic protozoan, may be an important cause of neurological and psychiatric diseases. The present systematic review and meta-analysis, therefore, was conducted to investigate the scientific evidence regarding the potential association between *T*. *gondii* infection and psychiatric disorders in Iran.

**Methods:**

We systematically reviewed articles from world-wide databases, including PubMed, Scopus, Science Direct, Web of Science, Google Scholar, and Iranian national databases up to July 30^th^, 2021. The Newcastle Ottawa Scale (NOS) was used to assess the quality of included studies. The common odds ratio (OR) was estimated using inverse variance and a random-effects model. Heterogeneity was assessed using the χ2-based Cochrane test (Q) and the *I*^*2*^ index. Also, sensitivity analyses and publication bias were calculated. Moreover, subgroup analysis was performed based on the type of disorder and quality score of different eligible studies.

**Results:**

16 studies were included in this meta-analysis. Our meta-analyses found that the OR of the risk of anti- *T*. *gondii* IgG and IgM in psychiatric patients compared to the control group was 1.56 (95% CI; 1.23–1.99) and 1.76 (95% CI: 1.19–2.61), respectively. Subgroup analysis based on the type of disorder showed that the OR of the risk of anti- *T*. *gondii* IgG in Iranian schizophrenia patients and other psychiatric disorders compared to the control group were 1.50 (95% CI; 1.09–2.07) and 2.03 (95% CI: 1.14–3.60), respectively, which are statistically significant. Also, the OR of the risk of anti- *T*. *gondii* IgM in Iranian schizophrenia and depression patients compared to the control group was 1.54 (95% CI; 0.9–2.64) and 1.03 (95% CI: 0.2–5.24), respectively, which are not statistically significant. Additionally, subgroup analysis based on quality scores showed no significant influence on the results according to the moderate quality studies. However, this association was significant according to the high quality studies. The obtained results of Egger’s test were 1.5 (95% CI; -0.62–3.73, *P* = 0.15) and 0.47 (95% CI; -0.82–1.76, *P* = 0.45), respectively, indicating publication bias. The significant results of the heterogeneity analysis confirmed a high level of heterogeneity in the IgG test (*P* = 0.000, *I*^*2*^ = 66.6%). However, no significant results from the test of heterogeneity were detected in the IgM test (*P* = 0.15, *I*^*2*^ = 27.5%). The results of the sensitivity analysis showed that the impact of each study on the meta-analysis was not significant on overall estimates.

**Conclusions:**

Despite the limited number of studies, these outcomes supported a possible link between *T*. *gondii* infection and psychiatric disorders in Iran. However, more high-quality investigations are needed in the future.

## Introduction

*Toxoplasma gondii* (*T*. *gondii*) is one of the most successful parasites in the world and is responsible for a neglected parasitic disease, toxoplasmosis, which forms tissue cysts in some tissues, including the brain of warm-blooded animals such as humans [1]. Human infection occurs in several ways, including by consumption of meat containing tissue cysts harboring bradyzoites and by ingestion of food or water contaminated with sporulated oocysts [2]. *T*. *gondii* prevalence in humans is different in developed and developing countries, and in some areas it can be high (e.g., Brazil, 77.5%; Sao Tome and Principe, 75.2%; Iran, 63.9%; Colombia, 63.5%; and Cuba, 61.8%) [3].

Tachyzoites, bradyzoites (the tissue cyst form), and sporozoites are three infectious forms of the *T*. *gondii* parasite. After the activation of the host’s immune system, some of the tachyzoites differentiate into slowly-growing bradyzoite forms, establishing themselves within tissue cysts, which initiates the chronic or latent phase of the disease [4]. These tissue cysts can be reactivated when the immune function is weak [5].

Toxoplasmosis is commonly asymptomatic in immunocompetent individuals, although prenatal infection with this ubiquitous parasitic protozoan may cause abortion as well as a congenital disease that includes severe intellectual disability and seizures [6]. Also, different investigations suggested that chronic *T*. *gondii* infection could be related to various psychiatric disorders and changes in the personality of individuals in humans and animal models [7–9].

These disorders caused by *T*. *gondii* may be the result of the parasite’s ability to infect a variety of brain cells, including neurons and microglial cells, causing death in host cells. In addition, IFN- is produced, which results in altered dopamine levels. Following *T*. *gondii* infection, a large amount of neurotransmitters, such as dopamine, are produced, which may be responsible for psychiatric disorders [10–12].

Results of previous systematic and meta-analysis studies have shown the association between *T*. *gondii* and some psychiatric disorders such as epilepsy, depression, bipolar disorder, and schizophrenia [13–17]. However, there has been no systematic review and meta-analysis describing the status of *T*. *gondii* and psychiatric disorders in Iran; hence, the current systematic review and meta-analysis was aimed to exploring the relationship between *T*. *gondii* and psychiatric disorders in this country.

## Methods

### Search strategy

This study was performed according to the Preferred Reporting Items for Systematic Reviews and Meta-Analysis (PRISMA) and its checklist (S1 Checklist) [18]. In this systematic review and meta-analysis we sought to determine the epidemiological aspects of toxoplasmosis in patients with psychiatric disorders in Iran. The search strategy was performed based on the online literature screening in five English databases (PubMed, Scopus, Science Direct, Web of Science and Google Scholar) and three Persian databases (Magiran, Irandoc and Idml) up to July 30^th^, 2021. This review was accomplished using medical subject heading (MeSH) terms in combination or alone: (*Toxoplasma gondii* OR Toxoplasmosis) AND (Mental Disorders OR Psychiatric Illness OR Psychiatric Disease OR Psychiatric Disorder OR Behavior Disorders) AND IRAN (S1 Table).

### Inclusion and exclusion criteria

The inclusion criteria included: (1) studies published in English or Persian until July 30^th^, 2021, (2) case-control and cross-sectional studies about the relationship between toxoplasmosis and psychiatric disorders, (3) original research papers, (4) original articles with available full texts in English and Persian languages, and (5) studies with information on the exact total sample size and positive samples in the case and control groups, (6) studies performed only on humans, (7) studies providing detailed information on the prevalence of toxoplasmosis using serology (i.e., IgG and/or IgM antibodies). The exclusion criteria were: (1) studies in other countries, expect Iran, (2) review articles, and (3) non-human studies.

### Study selection and data extraction

All the retrieved papers from the search strategy were imported to EndNote (version X7). After the removal of duplicate papers, the titles and abstracts were studied exactly by two different reviewers (EM and MM). The reviews, systematic review and meta-analysis, studies in other countries, non-serological studies, and animal experimental studies were excluded to assess the final eligibility and inclusion criteria. Then, the information about the name of the first author, year of publication, psychosomatic disorder, location of the study, type of study, method, type of antibody, and age and gender of Iranian patients with psychiatric disorders and controls was extracted into a Microsoft Excel datasheet.

### Quality assessment

In order to assess the quality of reporting of the studies, the Newcastle Ottawa Scale (NOS) with separate criteria for case-control and cross-sectional studies was used [19]. Based on the NOS checklist, in the case-control studies, the scores of 3, 4–6, and 7–9 were representative of low, moderate, and high quality, respectively. Furthermore, regarding the cross-sectional studies, the scores of 1–2, 3–5, and 6–7 indicated low, moderate, and high quality, respectively.

### Meta-analysis

The meta-analysis was executed with STATA version 11 (STATA Corp., College Station, Texas, USA). The common odds ratio (OR) was estimated using inverse variance and a random-effects model for each included study. To display the heterogeneity between studies, χ2-based Cochrane test (Q) and *I*^*2*^ index were applied [20]. The publication bias was examined by the Egger test [21]. Also, a sensitivity analysis was performed to identify the probable effect of each article on the overall results by excluding them using STATA software. Additionally, subgroup analysis was performed based on the type of disorder and quality score of different eligible studies.

## Results

**Fig 1:**
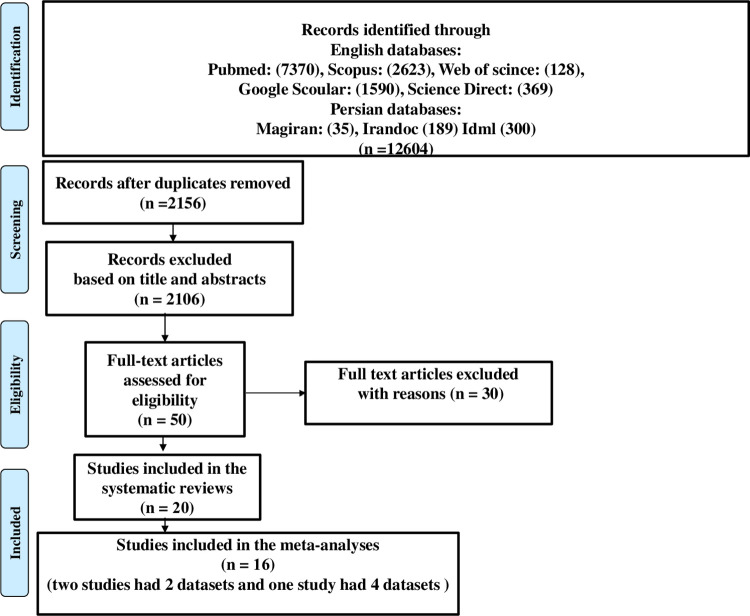
Flow diagram of the study design process.

Full texts of 20 articles had eligibility to be accounted for in the systematic review according to inclusion criteria, as shown in Fig 1. Out of 20 studies, a total of 4 papers [22–25] were excluded due to the lack of a control group, and eventually, 16 of these articles (two studies containing 2 datasets and one study containing 4 datasets) were entered into this meta-analysis with respect to the inclusion/exclusion criteria. Information and characteristics of each study in systematic review and meta-analysis are presented in Table 1 and S2 Table, respectively. The included studies in the present meta-analysis showed acceptable quality (i.e., ≥3 for cross-sectional studies and ≥4 for case-control studies). S3 Table shows the quality of the included studies.

**Table 1:** Characteristics of the included studies for *T*. *gondii* infection in Iranian psychiatric patients and controls.

No	First Author (Publication Year)	Psychiatric Disorder	City	Type of study	Method	Type of antibody	Age (years±SD)	Gender (N)	*T*. *gondii* IgG prevalence (%)	*T*. *gondii* IgM prevalence (%)	P-value
1	Gharavi MJ, 2005 [23]	Intellectual disability children	Tehran	Descriptive	ELISA, IFA	IgG and IgM	0–29	*T*. *gondii* Pos P: (F:22, M:27)	P: 13.89	P: 0	
2	Sharif M, 2007 [25]	Intellectual disability children	Sari	Descriptive	IFAT	IgG	5> 5–20 20≤	P: (F:175, M:161)	P: 77.4		
3	Saraei-Sahnesaraei M, 2009 [40]	Schizophrenia	Qazvin	Case control	ELISA	IgG and IgM	35/36± 12/65	P: (F:36, M:66) C: 114	P: 55.3 C: 50.9	P: 14.4 C: 20	
4	Daryani A, 2010 [41]	Schizophrenia	Sari	Case control	ELISA	IgM and IgG	P: 32.95±10.05 C: 33.76±10.50	-	P: 35 C: 25.3	P: 11.2 C: 11.1	
5	Hamidinejat H, 2010 [42]	Schizophrenia	Ahvaz	Case control	ELISA	IgM and IgG	18–58	-	P: 57.1 C: 29.2	P; 4.1 C: 4.3	<0.05^a^
6	Alipour A, 2011 [43]	Schizophrenia	Tehran	Case control	ELISA	IgG	P: 37.54±9.75 C: 37.24±10.24	P: (F:23, M:39) C: (F:36, M:26)	P: 67.7 C: 37.1		<0.01^a^
7	Khalili B, 2014 [44]	Intellectual disability	Chahrmahal Va Bakhtiari	Case control	ELISA	IgM and IgG	-	P: (F:22, M:86) C: 50	P: 31 C: 14	P: 7 C: 1	
8	Khademvatan Sh, 2014 [45]	Schizophrenia	Ahvaz	Case control	ELISA	IgM and IgG	20> 20–50 50<	P: (F:35, M:65) C: (F:43, M:51)	P: 34 C: 47.39	P: 4 C: 2.1	
9	Ebadi M, 2014 [46]	Schizophrenia	Tehran	Case control	ELISA	IgG and IgM	P: 20–64 C: 19–84	P: (F:67, M:85) C: 152	P: 53.2 C: 41.4	P: 58.5 C: 47.6	0.03^a^ 0.01^b^
10	Khademvatan Sh, 2014 [47]	Schizophrenia	Ahvaz	Case control	ELISA	IgG	20> 20–50 50<	P: (F:35, M:65) C: (F:104, M:96)	P: 34 C: 26.5		
11	Ezatpour B, 2015 [22]	Intellectual disability	Lorestan	Descriptive	ELISA	IgG	10–30	P: (F:64, M:94)	P: 30.4		
12	Nourollahpour Shiadeh M, 2016 [48]	Prenatal depression in pregnant women	Tehran	Case control	ELISA	IgG	P: 28.9 ± 5.6 C: 28.2 ±5.4	P: (F:116) C: (F:244)	P: 59.5 C: 51.2		
13	Kheirandish F, 2016 [49]	Mood disorders	Lorestan	Case control	ELISA	IgG and IgM	25> 25–50 50<	P: (F:73, M:97) C: (F:67, M:103)	P: 60.5 C: 38.2	P: 6.5 C: 2.9	0.009^a^
14	Afsharpaiman Sh, 2016 [50]	Hyperactivity disorder	Tehran	Case control	ELISA	IgG and IgM	P: 8.12± 3.25 C: 8.12± 2.40	P: (F:16, M:32) C: (F:16, M:32	P: 4.2 C: 2.1	P: 2.1 C: 0	
15	Afsharpaiman Sh, 2017 [51]	Anxiety disorders (OCD or phobias)	Tehran	Case control	ELISA	IgG and IgM	P:8.56±2.5 C:8.42±1.9	P: (F:25, M:23) C: (F:25, M:23)	P: 2.1 C: 2.1	P: 0 C: 2.1	
16	Abdollahian E, 2017 [52]	Schizophrenia, bipolar, mental and other disorders	Mashhad, Northeast of Iran	Case control	ELISA	IgG and IgM	10–80	P: (F:87, M:263) C: (F:180, M:170)	P: 46.85 C: 34.28	P: 4.85 C: 0.85	
17	Ansari-Lari M, 2017 [53]	Schizophrenia	Shiraz	Case control	ELISA	IgG	P: 40.3±10.2 C: 40.6±10.7	P: (F:27, M:72) C: (F:42, M:110)	P: 42 C: 27		0.012^a^
18	Anoshirvani K, 2019 [54]	Mental Disorder	Tehran	Cross sectional	ELISA	IgG	18–25	M: 239	P: 28.87		
19	Nasirpour S, 2020 [55]	Depression	Khorramabad	Case control	ELISA PCR	IgM and IgG	≥30 30–60 60<	P: (F:38, M:49) C: (F:61, M:26)	P: 59.8 C: 56.3	P: 0 C: 0	
20	Mirahmadi H, 2020 [24]	Schizophrenia	Zahedan and Zabol	Cross sectional	ELISA LAMP assay Nested-PCR	IgM and IgG	19–55	P: (F:19, M:99)	P: 55.9	P: 3.37	

ELISA: Enzyme-linked immunosorbent assay, IFA: Indirect immunofluorescence assay, Nested-PCR: Nested-polymerase chain reaction, IgG: Immunoglobulin G, IgM: Immunoglobulin M, P: Patient, C: Control, F: Female, M: Male.

^a^A significant difference in *T*. *gondii* IgG prevalence between the case and control groups.

^b^A significant difference in *T*. *gondii* IgM prevalence between the case and control groups.

Out of 20 studies, a total of 10 papers evaluated the *T*. *gondii* infection in Iranian schizophrenia patients. The investigated articles were from different cities in Iran, such as Tehran (7 studies), Ahvaz (3 studies), Sari and Lorestan (2 studies), Qazvin, Chahrmahal Va Bakhtiari, Mashhad, Shiraz, Khorramabad, Zahedan, and Zabol (1 study).

The enzyme-linked immunosorbent assay (ELISA) was the most commonly used method to detect anti-*T*. *gondii* IgG and IgM antibodies in studies. 7 and 13 studies addressed anti-*T*. *gondii* IgG, and both IgG and IgM, respectively.

**Fig 2:**
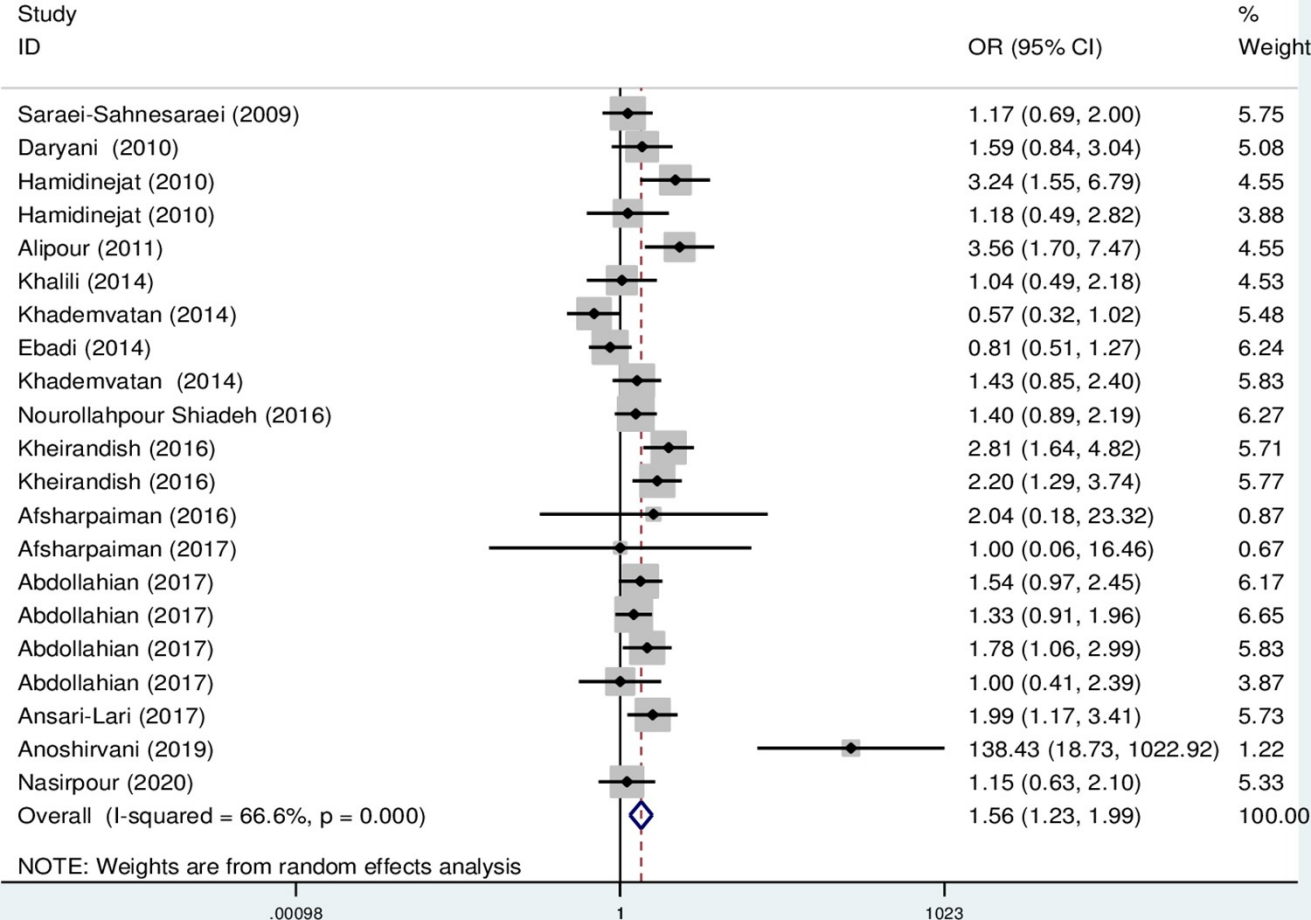
Forest plot diagram of studies showing IgG seropositivity rate of *T*. *gondii*.

**Fig 3:**
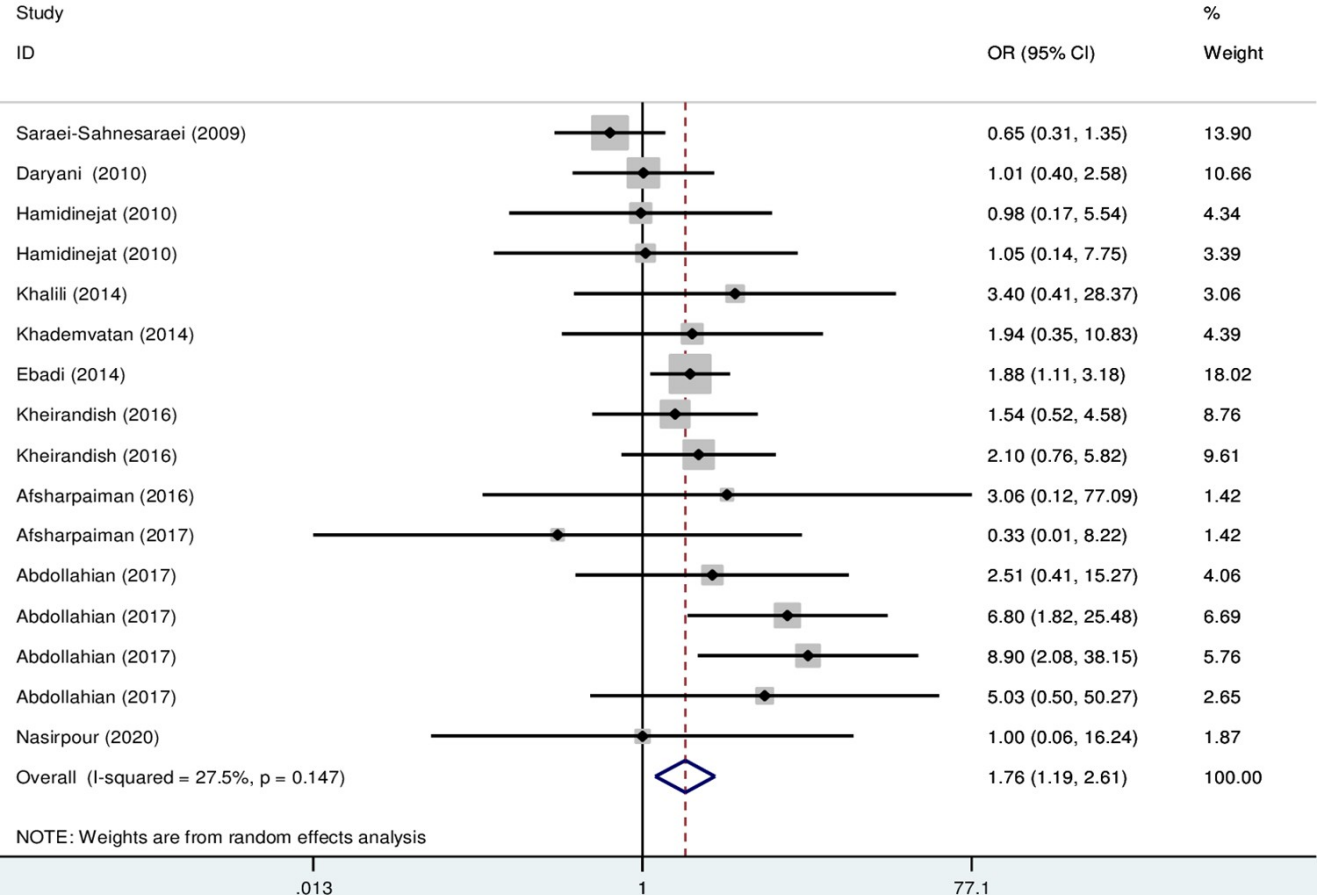
Forest plot diagram of studies showing IgM seropositivity rate of *T*. *gondii*.

The results of the meta-analysis indicate that the OR of the risk of anti- *T*. *gondii* IgG and IgM in psychiatric patients compared to the control group was 1.56 (95% CI; 1.23–1.99) and 1.76 (95% CI: 1.19–2.61), respectively, which are statistically significant (Figs 2 and 3).

**Fig 4:**
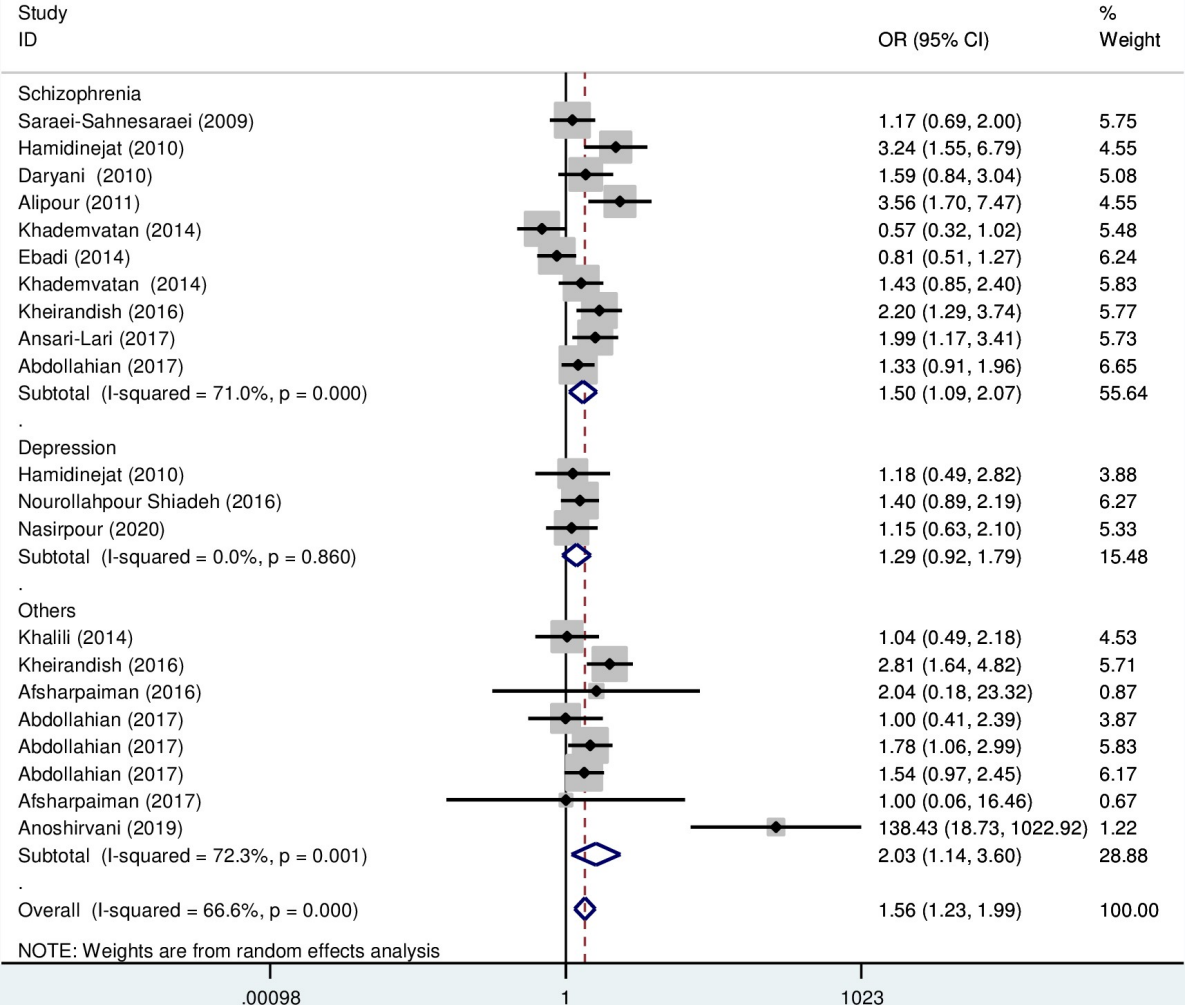
Forest plot diagram of studies showing IgG seropositivity rate of *T*. *gondii* based on the type of disorder.

**Fig 5:**
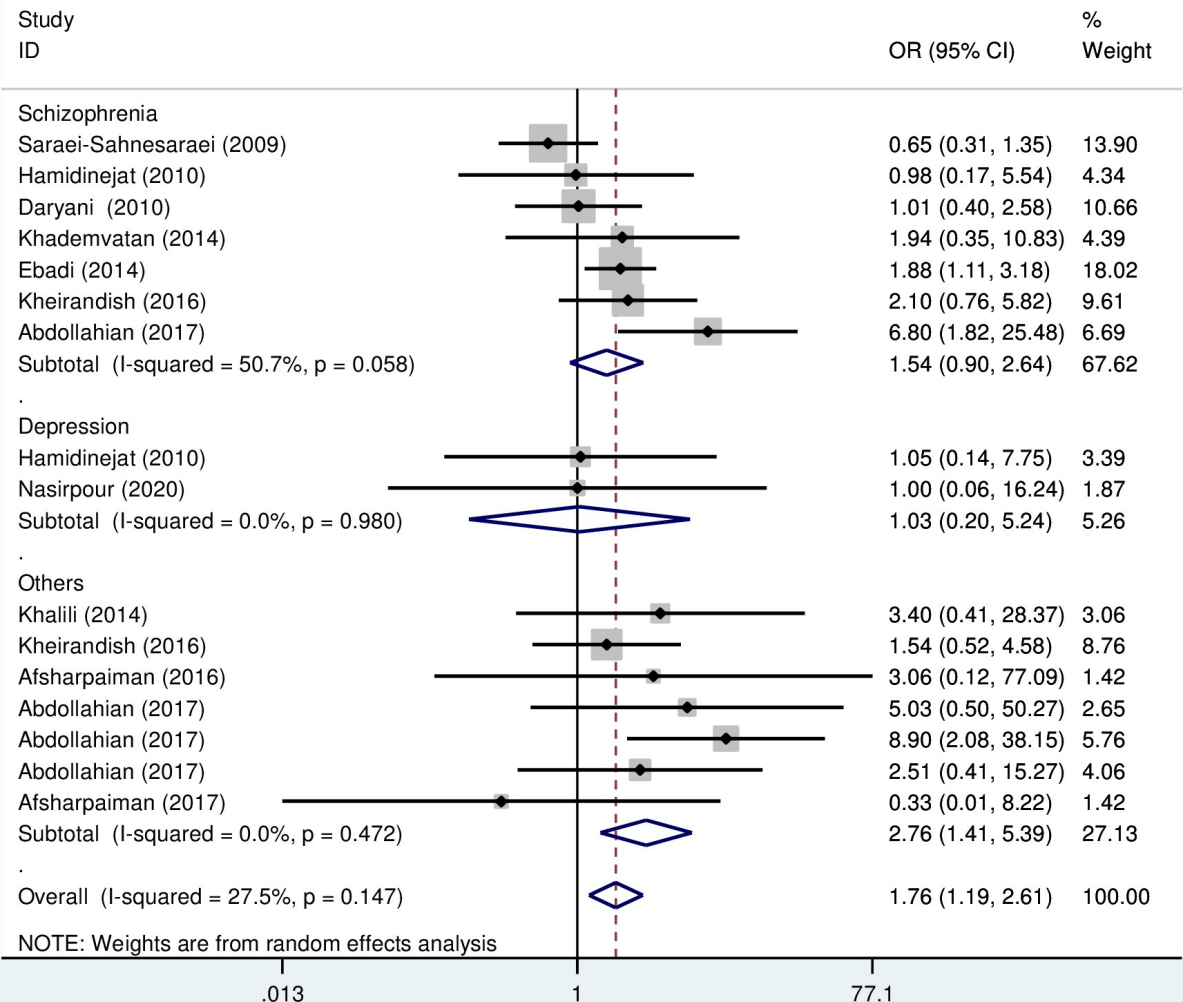
Forest plot diagram of studies showing IgM seropositivity rate of *T*. *gondii* based on the type of disorder.

**Fig 6:**
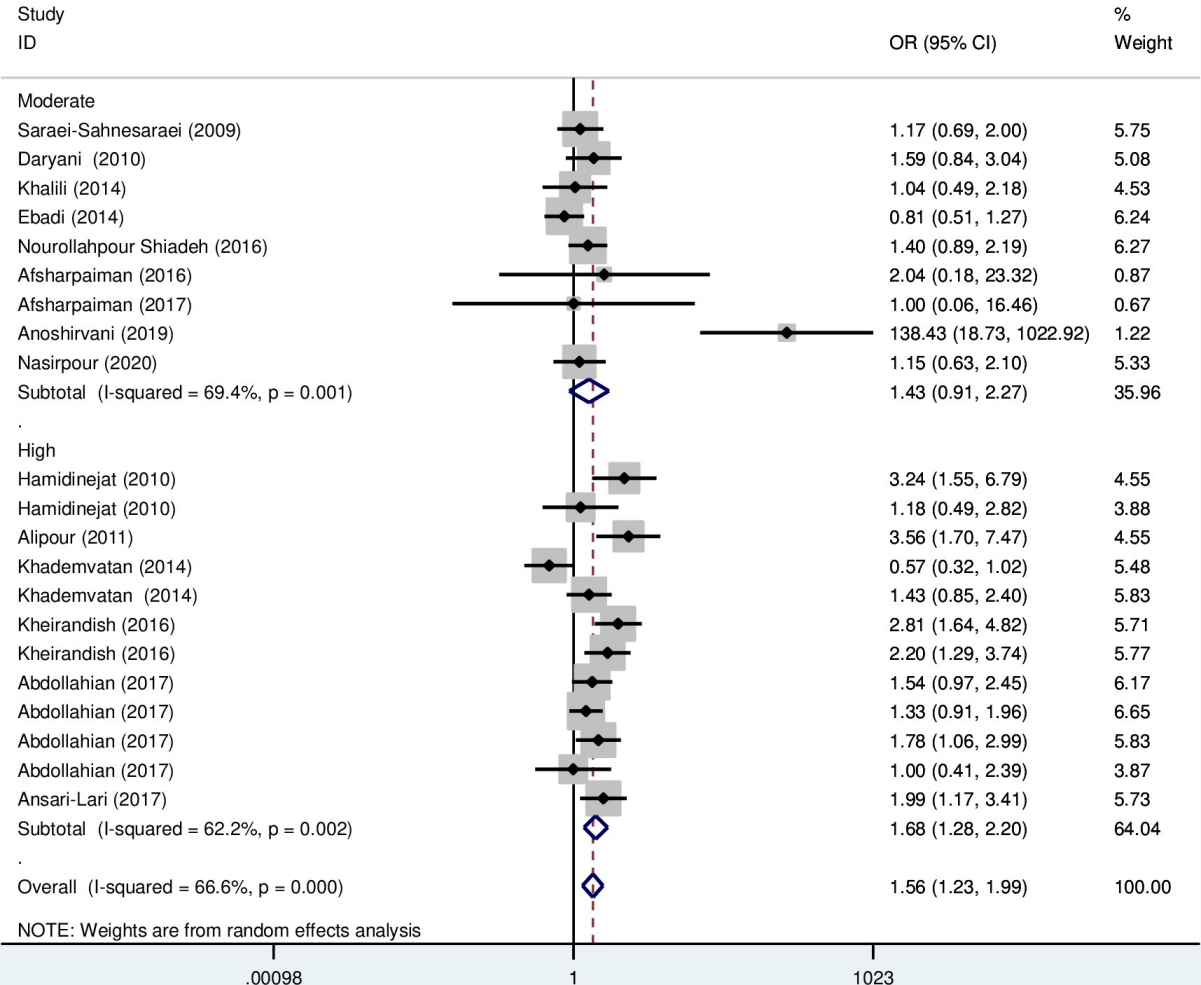
Forest plot diagram of studies showing IgG seropositivity rate of *T*. *gondii* based on the quality score of different eligible studies.

**Fig 7:**
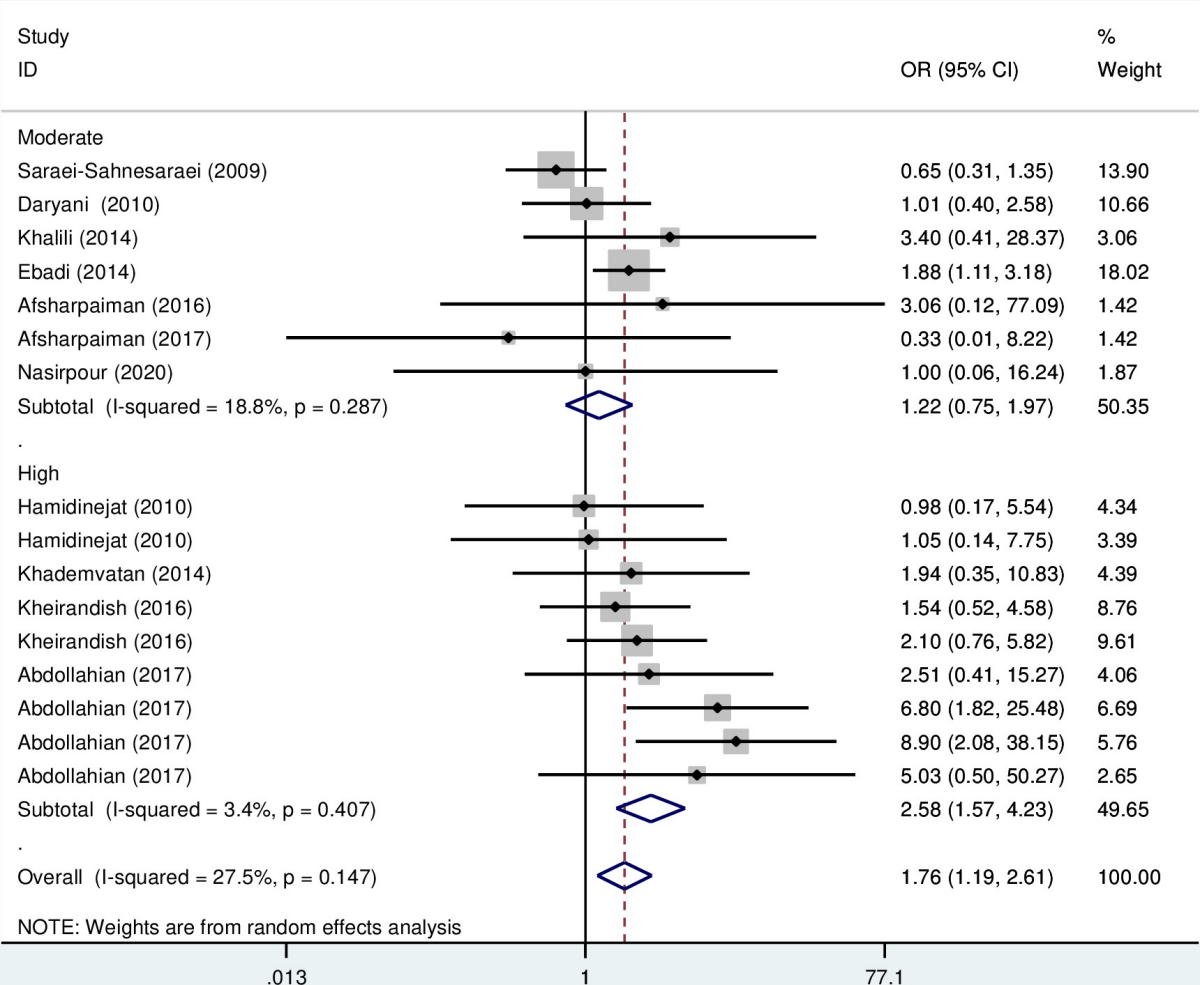
Forest plot diagram of studies showing IgM seropositivity rate of *T*. *gondii* based on the quality score of different eligible studies.

Subgroup analysis based on the type of disorder showed that the OR of the risk of anti- *T*. *gondii* IgG in Iranian schizophrenia patients and other psychiatric disorders compared to the control group were 1.50 (95% CI; 1.09–2.07) and 2.03 (95% CI: 1.14–3.60), respectively, which are statistically significant (Fig 4). Also, the OR of the risk of anti- *T*. *gondii* IgM in Iranian schizophrenia and depression patients compared to the control group was 1.54 (95% CI; 0.9–2.64) and 1.03 (95% CI: 0.2–5.24), respectively, which are not statistically significant (Fig 5). Additionally, subgroup analysis based on quality scores of different eligible studies showed that the OR of the risk of anti- *T*. *gondii* IgG in other psychiatric patients compared to the control group was 1.68 (95% CI; 1.28–2.20) and 1.43 (95% CI; 0.91–2.27), respectively (Fig 6). Also, the OR of the risk of anti- *T*. *gondii* IgM in psychiatric patients compared to the control group based on the high and moderate quality studies were 2.58 (95% CI; 1.57–4.23) and 1.22 (95% CI; 0.75–1.97), respectively (Fig 7).

**Fig 8:**
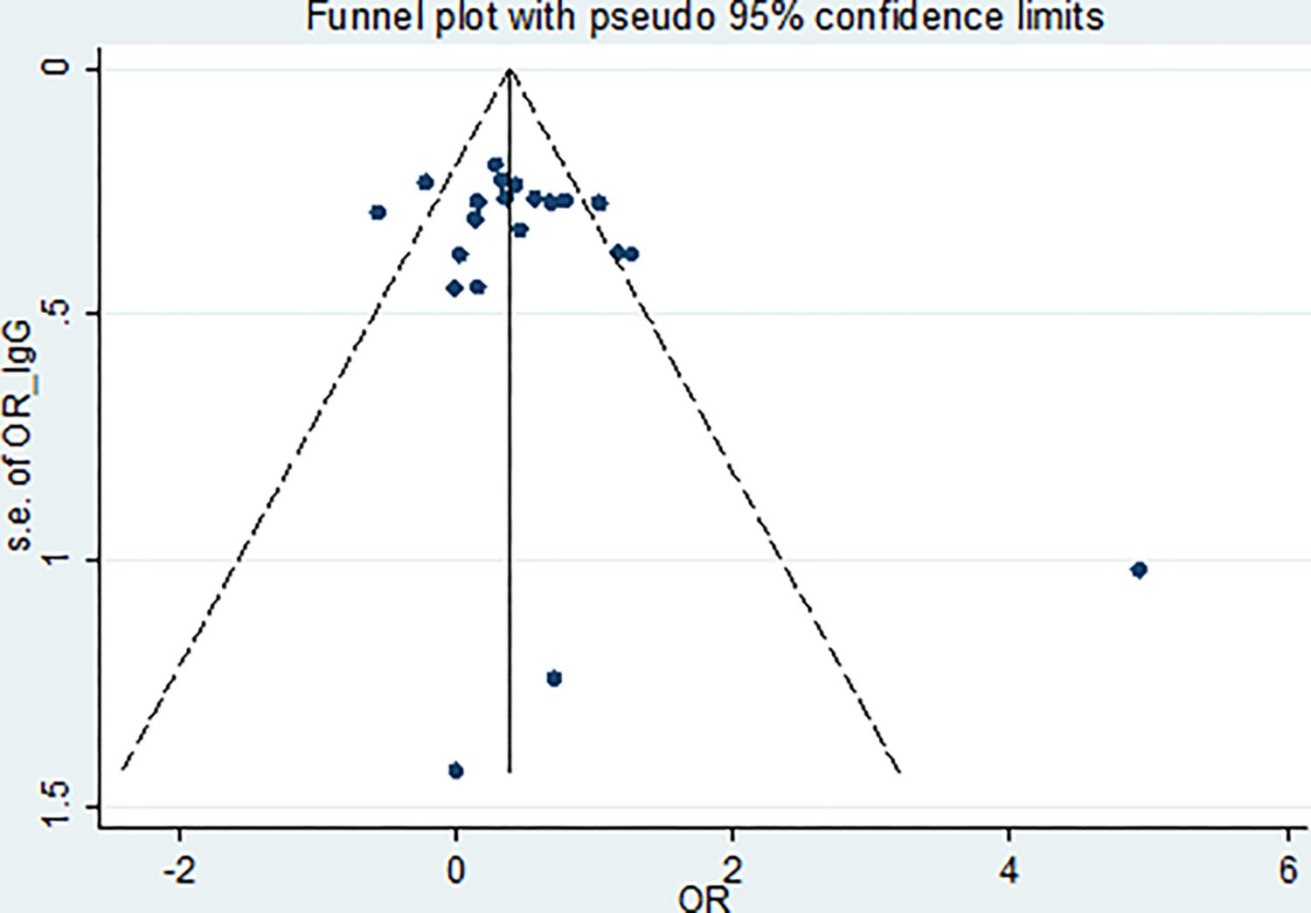
Funnel plot for detecting publication bias based on IgG.

**Fig 9:**
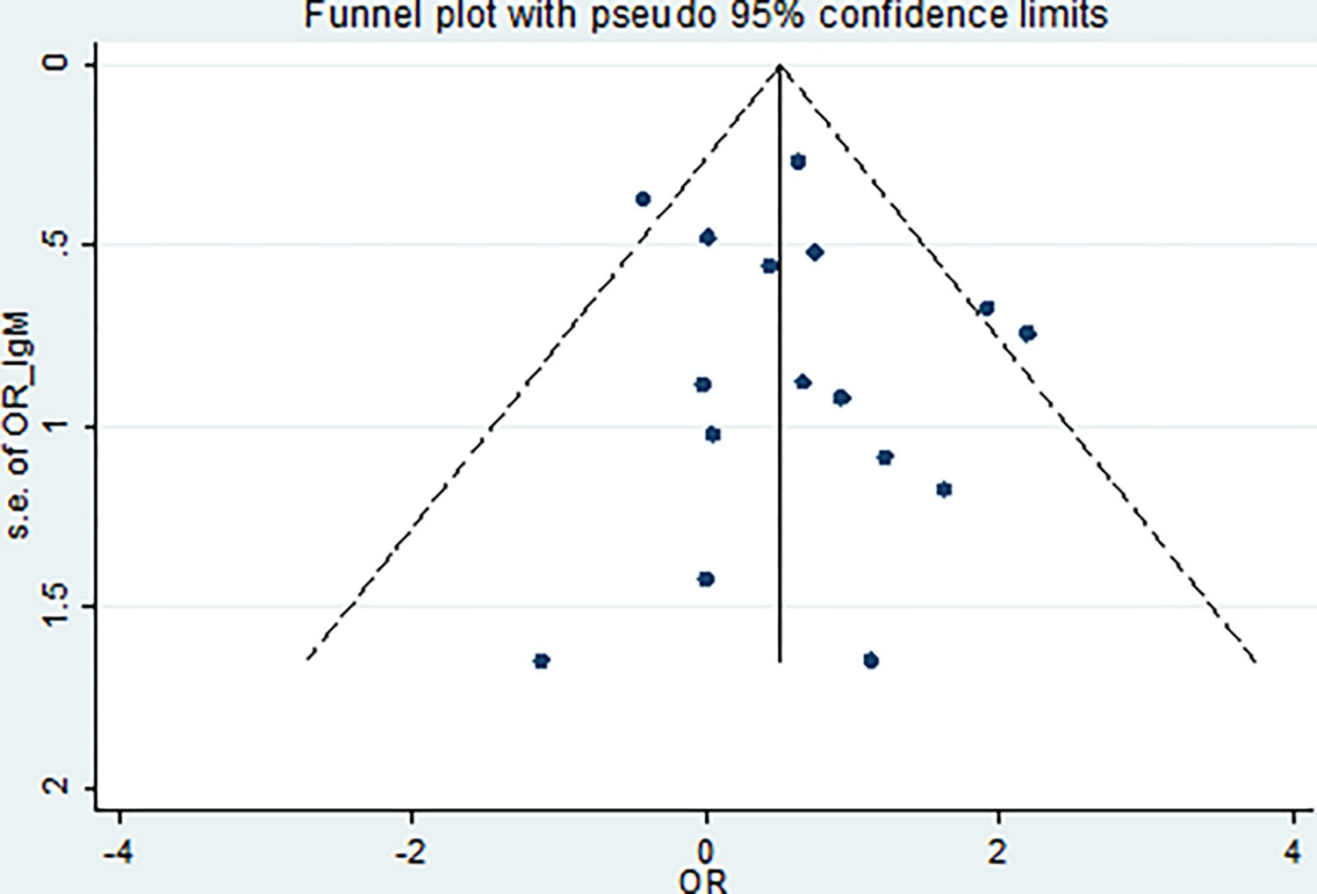
Funnel plot for detecting publication bias based on IgM.

The obtained results of Egger’s test were 1.5 (95% CI; -0.62–3.73, *P* = 0.15) and 0.47 (95% CI; -0.82–1.76, *P* = 0.45), respectively, indicating publication bias (Figs 8 and 9). The significant results of the heterogeneity analysis confirmed a high level of heterogeneity in the IgG test (*P* = 0.000, *I*^*2*^ = 66.6%). However, no significant results from the test of heterogeneity were detected in the IgM test (*P* = 0.15, *I*^*2*^ = 27.5%).

**Fig 10:**
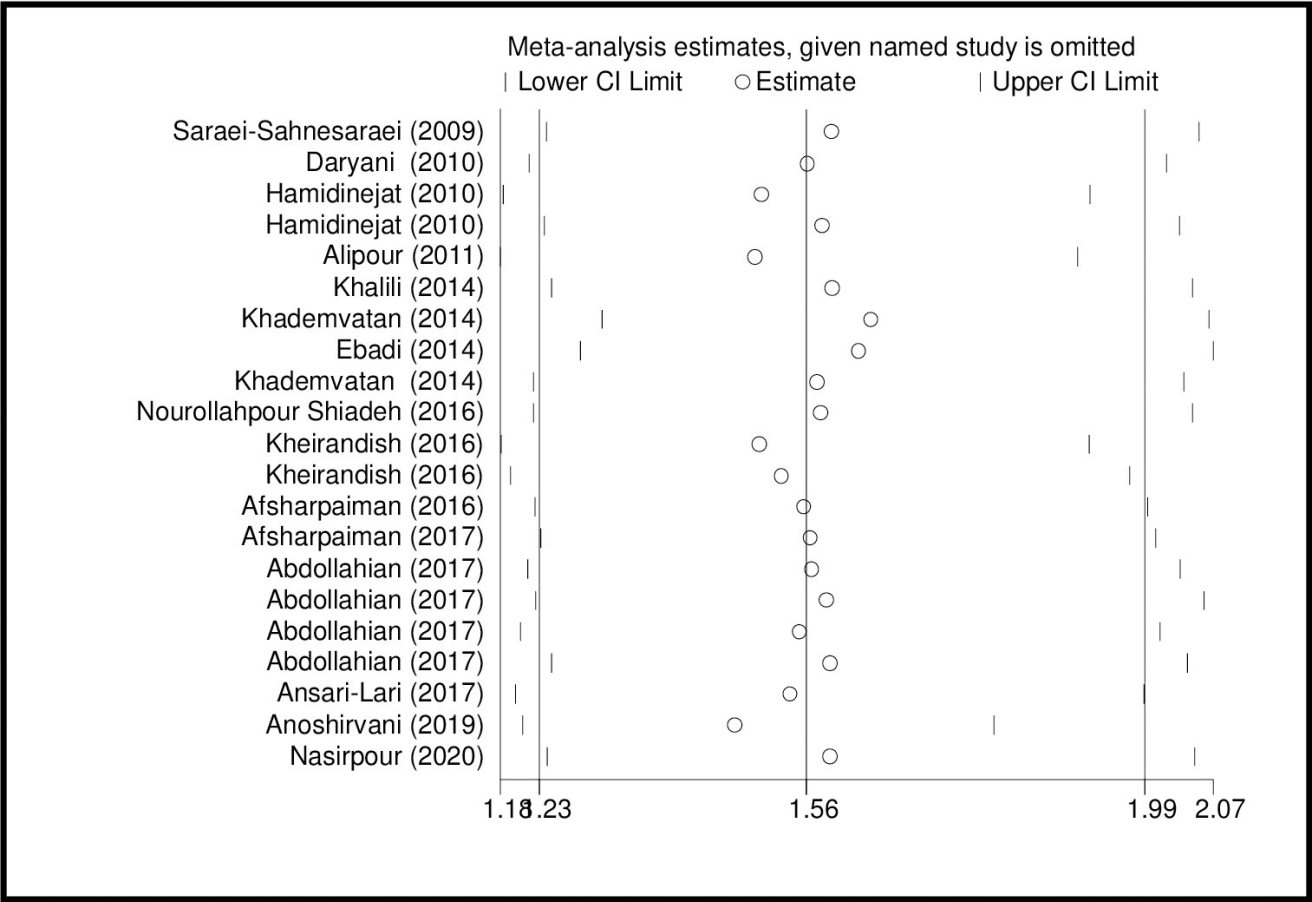
Sensitivity analysis based on IgG for assessing the effect of each primary study on the total estimates.

**Fig 11:**
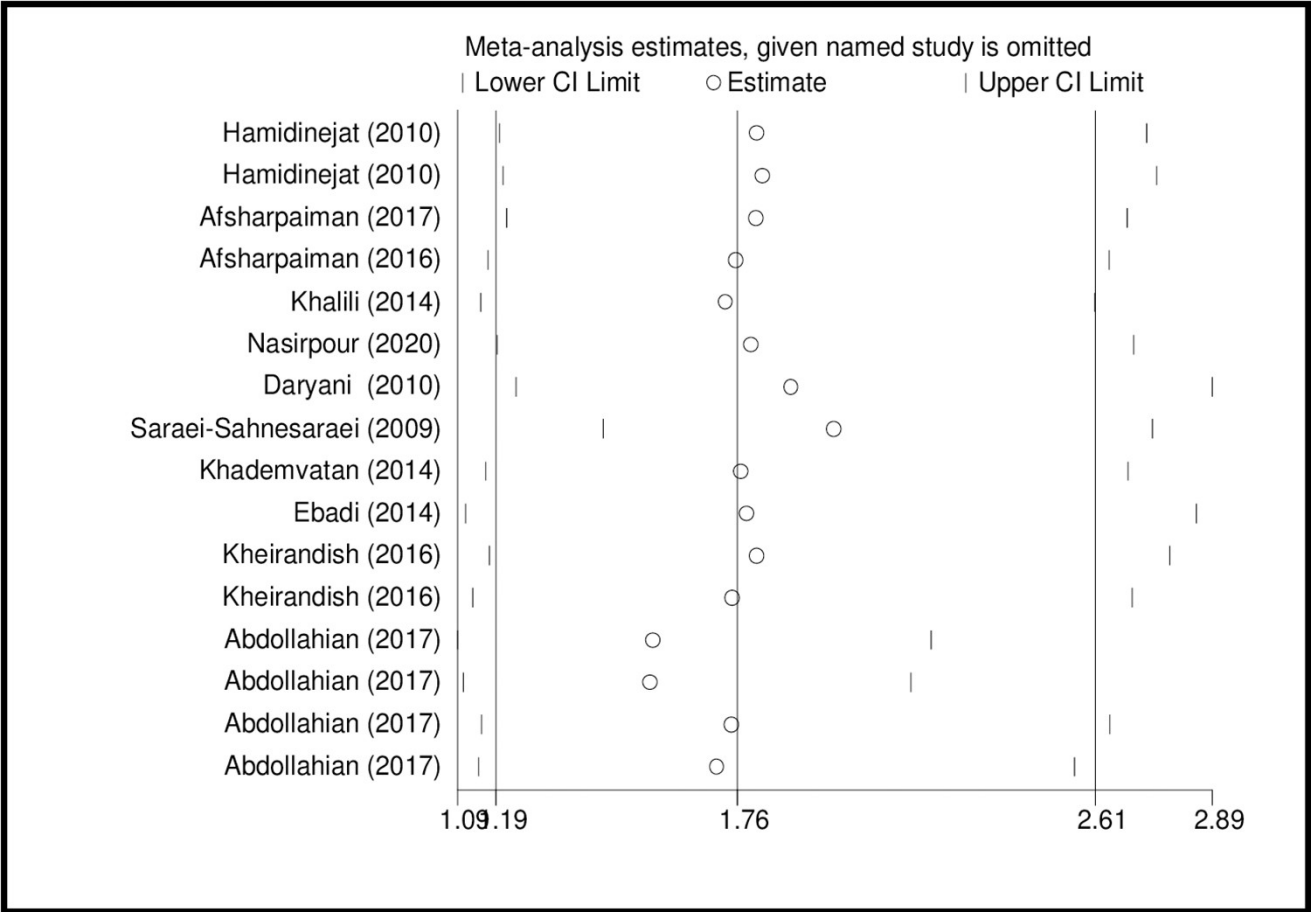
Sensitivity analysis based on IgM for assessing the effect of each primary study on the total estimates.

The results of sensitivity analysis showed that the impact of each study on the meta-analysis was not significant on overall estimates (Figs 10 and 11).

## Discussion

Nowadays, investigation on the correlation between *T*. *gondii* infection and psychiatric disorders has become a hot topic [26]. This systematic review and meta-analysis aimed to quantify the pooled ORs of anti-*T*. *gondii* IgG and IgM antibodies in Iranian psychiatric patients and compare them with those of control groups in Iran. The results of the present study indicated a significantly higher *T*. *gondii* IgG and IgM in the psychiatric patients compared to those in the control group, with an OR of 1.56 (95% CI; 1.23–1.99) and 1.45 (95% CI: 1.10–1.92). Based on these results, toxoplasmosis can be considered a risk factor for psychiatric patients, and thus, these patients should pay attention to the detection of *T*. *gondii* in this area.

Based on subgroup analysis, the OR of the risk of anti- *T*. *gondii* IgG in Iranian schizophrenia and other psychiatric disorder patients was > 1 and significant. However, this connection between latent toxoplasmosis and depression was not significant. Given that few studies have reported a link between certain psychiatric disorders (bipolar, mental, anxiety, hyperactivity, and intellectual disability) and *Toxoplasma* infection in Iran, a subgroup analysis based on the type of disorder was performed in tandem. In addition, the small number of studies limits the reliability of results, and more investigation are required to shed more light on this line. Similar to our results, several meta-analysis studies have reported a positive association between *T*. *gondii* infection with a higher IgG level and the presence of schizophrenia [14–16, 27]. Thus, *T*. *gondii* infection can be considered an underlying component of the pathophysiology of schizophrenia [26]. Also, similar to our results a meta-analysis by Cossu et al. which consisted of a total of 4021 patients with bipolar disorder and 8669 healthy control people in 23 studies, recently revealed that the prevalence of IgG class antibodies to *T*. *gondii* in patients with bipolar disorder was significantly higher than the prevalence of antibodies in control groups [28].

Contradictory results have been reported regarding the possible link between *T*. *gondii* infection and depression. However, despite the limited number of studies, a relationship between *T*. *gondii* seropositivity and depression cannot be confirmed. Our findings are consistent with the findings of the two previous meta-analyses [14, 29].

According to our subgroup analysis, a significant association of *T*. *gondii* IgM antibodies and Iranian schizophrenia and depression patients was not observed. Based on these results, acute toxoplasmosis is not considered a risk factor for these patients. However, the prevalence of *T*. *gondii* IgM in patients with other psychiatric disorders was significantly higher than the prevalence of IgM in control groups, with a combined OR of 2.76 (95% CI: 1.41–5.39). In the meta-analysis performed by Sutterland et al., on the association of *T*. *gondii* infection with recent onset schizophrenia, a non-significant OR of 1.24 (95% CI 0.98–1.57, *P* = 0.08) was found [14]. In return, Monroe et al., in a meta-analysis reported a significant increase in the risk of positive *T*. *gondii* IgM antibodies in acute psychosis in patients with schizophrenia compared with controls [30]. IgM is an indicator of an acute or recent infection or a potentially persistent infection or reinfection, probably with a different genotype of *T*. *gondii* [31–34]. These variations in these findings might be related to sociocultural habits, geographical and environmental factors, sample size, and methodology in the studied population [35–37]. Moreover, there is evidence that different genotypes of *T*. *gondii* have diverse effects on the course of psychosis [38]. The study of these variables aid in improving the precision of future studies describing the relationship between infectious agents and psychiatric disorders [39]. However, due to different age ranges and gender proportions of participants and the almost identical methods in the selected studies (ELISA), subgroup analysis based on these two variables was not performed in this study. These variables may have affected the outcomes of our meta-analysis. Additionally, we found no significant influence on the results from subgroup analysis according to the moderate quality studies based on anti- *T*. *gondii* IgG and IgM in psychiatric patients. However, this association was significant according to the high quality studies. A higher score on the NOS checklist indicates a higher quality of a study. In fact, the quality of the majority of the effective items was taken into account in these studies.

Furthermore, we found high heterogeneity in the association between psychiatric disorders and *T*. *gondii* infection in this study. There are several sources of heterogeneity. In case-control studies, selection of cases and controls in different populations and in different ways has been done. Moreover, different ways of selecting the target groups can be a source of heterogeneity. The age of the participants in the studies is another source of heterogeneity because advanced age increases the chance of exposure to *Toxoplasma*.

The included studies in our meta-analysis study were from 10 different cities in Iran. However, data gaps were identified for other cities where no data was available on the association between *T*. *gondii* and psychiatric disorders. Therefore, it is highly necessary to find experimental documents from other parts of Iran.

## Conclusions

In conclusion, to the best of our knowledge, this is the first systematic review and meta-analysis providing a general view of a possible link between *T*. *gondii* and psychiatric disorders in Iran. However, many questions remained to be answered in future investigations. Also, further research should be performed to investigate into measures that could decrease the high prevalence of *Toxoplasma* in Iran.

## Supporting information

S1 Checklist PRISMA 2009 checklist. (DOC)

S1 Table Complete list of terms used for database search. (DOC)

S2 Table Characteristics of the included studies for *T*. *gondii* serological analysis in cases (Iranian psychiatric patients) and controls. (DOC)

S3 Table Quality assessment of included studies based on the Newcastle-Ottawa Scale (NOS). (DOCX)
