# Supplementary material for: Relationship between Toxoplasma gondii infection and psychiatric disorders in Iran: A systematic review with meta-analysis
Source: PLoS One. 2023 Aug 8;18(8):e0284954. doi: 10.1371/journal.pone.0284954 (PMC10409283; doi:10.1371/journal.pone.0284954)
Supplement: S1 Table — (DOC) [file pone.0284954.s002.doc]

**Table S1. Complete list of terms used for database search.**

| Search engine | MeSH terms | Results (N) |
| --- | --- | --- |
| Pubmed | (((((((Toxoplasma gondii) OR (Toxoplasmosis)) AND (Mental Disorders)) OR (Psychiatric Illness)) OR (Psychiatric Disease)) OR (Psychiatric Disorder)) OR (Behavior Disorder)) AND (IRAN) | 7370 |
| Google scholar | (Toxoplasma gondii OR Toxoplasmosis) AND (Mental Disorders OR Psychiatric Illness OR Psychiatric Disease OR Psychiatric Disorder OR Behavior Disorders) AND IRAN | 1,590 |
| Science direct | (Toxoplasma gondii OR Toxoplasmosis) AND (Mental Disorders OR Psychiatric Illness OR Psychiatric Disease OR Psychiatric Disorder OR Behavior Disorders) AND IRAN | 369 |
| Scopus | (Toxoplasma gondii OR Toxoplasmosis) AND (Mental Disorders OR Psychiatric Illness OR Psychiatric Disease OR Psychiatric Disorder OR Behavior Disorders) AND IRAN | 2,623 |
| Web of Science | (Toxoplasma gondii OR Toxoplasmosis) AND (Mental Disorders OR Psychiatric Illness OR Psychiatric Disease OR Psychiatric Disorder OR Behavior Disorders) AND IRAN | 128 |
| Magiran | Toxoplasma gondii Mental Disorders  Toxoplasma gondii Psychiatric Illness  Toxoplasma gondii Psychiatric Disease  Toxoplasma gondii Psychiatric Disorder  Toxoplasma gondii Behavior Disorders  Toxoplasmosis Mental Disorders  Toxoplasmosis Psychiatric Illness  Toxoplasmosis Psychiatric Disease  Toxoplasmosis Psychiatric Disorder  Toxoplasmosis Behavior Disorders | 2  2  5  7  3  2  1  4  5  4 |
| Irandoc | Toxoplasma gondii  Toxoplasmosis | 113  76 |
| Idml | Toxoplasma gondii OR Toxoplasmosis AND Mental Disorders OR Psychiatric Illness OR Psychiatric Disease OR Psychiatric Disorder OR Behavior Disorders | 300 |
