# Supplementary material for: Relationship between Toxoplasma gondii infection and psychiatric disorders in Iran: A systematic review with meta-analysis
Source: PLoS One. 2023 Aug 8;18(8):e0284954. doi: 10.1371/journal.pone.0284954 (PMC10409283; doi:10.1371/journal.pone.0284954)
Supplement: S2 Table — (DOC) [file pone.0284954.s003.doc]

**Table S2.** Characteristics of the included studies for *T. gondii* serological analysis in cases (Iranian psychiatric patients) and controls.

| **ID** | **First author** | **Publication year** | **Type of disorder** | **N_case** | **N_positive**  **case_IgG** | **N_positive**  **case_IgM** | **N_control** | **N_positive**  **control_IgG** | **N_positive**  **control_IgM** | **Scores** |
| --- | --- | --- | --- | --- | --- | --- | --- | --- | --- | --- |
| 1 | Saraei-Sahnesaraei M | 2009 | Schizophrenia | 104 | 57 | 14 | 114 | 58 | 22 | 5 |
| 2 | Daryani A | 2010 | Schizophrenia | 80 | 28 | 9 | 99 | 25 | 11 | 5 |
| 3 | Hamidinejat H | 2010 | Schizophrenia | 98 | 56 | 4 | 48 | 14 | 2 | 8 |
| 3 | Hamidinejat H | 2010 | Depression | 46 | 15 | 2 | 48 | 14 | 2 | 8 |
| 4 | Alipour A | 2011 | Schizophrenia | 62 | 42 |  | 62 | 23 |  | 7 |
| 5 | Khalili B | 2014 | Intellectual disability | 108 | 31 | 7 | 50 | 14 | 1 | 5 |
| 6 | Khademvatan S | 2014 | Schizophrenia | 100 | 34 | 4 | 95 | 45 | 2 | 7 |
| 7 | Ebadi M | 2014 | Schizophrenia | 152 | 81 | 48 | 152 | 89 | 30 | 6 |
| 8 | Khademvatan S | 2014 | Schizophrenia | 100 | 34 |  | 200 | 53 |  | 8 |
| 9 | Nourollahpour Shiadeh M | 2016 | Depression | 116 | 69 |  | 244 | 125 |  | 6 |
| 10 | Kheirandish F | 2016 | Bipolar | 85 | 54 | 6 | 170 | 65 | 8 | 7 |
| 10 | Kheirandish F | 2016 | Schizophrenia | 85 | 49 | 8 | 170 | 65 | 8 | 7 |
| 11 | Afsharpaiman S | 2016 | Hyperactivity | 48 | 2 | 1 | 48 | 1 | 0 | 6 |
| 12 | Afsharpaiman S | 2017 | Anxiety | 48 | 1 | 0 | 48 | 1 | 1 | 6 |
| 13 | Abdollahian E | 2017 | Others | 94 | 41 | 2 | 350 | 117 | 3 | 7 |
| 13 | Abdollahian E | 2017 | Schizophrenia | 162 | 65 | 9 | 350 | 117 | 3 | 7 |
| 13 | Abdollahian E | 2017 | Bipolar | 70 | 33 | 5 | 350 | 117 | 3 | 7 |
| 13 | Abdollahian E | 2017 | Mental | 24 | 8 | 1 | 350 | 117 | 3 | 7 |
| 14 | Ansari-Lari M | 2017 | Schizophrenia | 99 | 42 |  | 152 | 41 |  | 7 |
| 15 | Anoshirvani K | 2019 | Mental | 124 | 68 |  | 115 | 1 |  | 4 |
| 16 | Nasirpour S | 2020 | Depression | 87 | 52 | 0 | 87 | 49 | 0 | 6 |

N: Number
