# Supplementary material for: Relationship between Toxoplasma gondii infection and psychiatric disorders in Iran: A systematic review with meta-analysis
Source: PLoS One. 2023 Aug 8;18(8):e0284954. doi: 10.1371/journal.pone.0284954 (PMC10409283; doi:10.1371/journal.pone.0284954)
Supplement: S3 Table — (DOCX) [file pone.0284954.s004.docx]

**Table S3**

Quality assessment of included studies based on the Newcastle-Ottawa Scale (NOS)

| No | First author | Publication year | Type of study | Selection | Comparability | Outcome | Total (score) |
| --- | --- | --- | --- | --- | --- | --- | --- |
| 1 | Saraei-Sahnesaraei M | 2009 | Case control | 2 | 1 | 2 | 5 |
| 2 | Daryani A | 2010 | Case control | 2 | 1 | 2 | 5 |
| 3 | Hamidinejat H | 2010 | Case control | 3 | 2 | 3 | 8 |
| 4 | Alipour A | 2011 | Case control | 3 | 2 | 3 | 7 |
| 5 | Khalili B | 2014 | Case control | 2 | 1 | 2 | 5 |
| 6 | Khademvatan Sh | 2014 | Case control | 2 | 2 | 3 | 7 |
| 7 | Ebadi M | 2014 | Case control | 2 | 2 | 2 | 6 |
| 8 | Khademvatan Sh | 2014 | Case control | 3 | 2 | 3 | 8 |
| 9 | Nourollahpour Shiadeh M | 2016 | Case control | 2 | 2 | 2 | 6 |
| 10 | Kheirandish F | 2016 | Case control | 3 | 2 | 2 | 7 |
| 11 | Afsharpaiman Sh | 2016 | Case control | 2 | 2 | 2 | 6 |
| 12 | Afsharpaiman Sh | 2017 | Case control | 2 | 2 | 3 | 7 |
| 13 | Abdollahian E | 2017 | Case control | 2 | 2 | 2 | 6 |
| 14 | Ansari-Lari M | 2017 | Case control | 2 | 2 | 3 | 7 |
| 15 | Anoshirvani K | 2019 | Cross sectional | 2 | 0 | 2 | 4 |
| 16 | Nasirpour S | 2020 | Case control | 2 | 2 | 2 | 6 |

High quality (7 – 9), moderate quality (4 – 6), and low quality (≤3) in case-control studies and high quality (6 and 7), moderate quality (3 – 5), and low quality (1 and 2) in cross sectional studies.
